# Axonal Type III Nrg1 Controls Glutamate Synapse Formation and GluA2 Trafficking in Hippocampal-Accumbens Connections

**DOI:** 10.1523/ENEURO.0232-16.2017

**Published:** 2017-02-27

**Authors:** Chongbo Zhong, Wendy Akmentin, Chuang Du, Lorna W. Role, David A. Talmage

**Affiliations:** 1 Department of Neurobiology and Behavior, Center for Nervous System Disorder, Stony Brook University, Stony Brook, NY 11794; 2 Department of Neuroscience, Tufts University School of Medicine, Boston, MA 02111; 3 Department of Pharmacological Science, Center for Nervous System Disorder, Stony Brook University, Stony Brook, NY 11794

**Keywords:** electron microscopy, glutamatergic transmission, neuregulin 1, neurotransmitter release, presynaptic maturation, synaptic vesicle fusion

## Abstract

Altered neuregulin 1 (Nrg1)/ErbB signaling and glutamatergic hypofunction have been implicated in the pathophysiology of schizophrenia. Here, we employed gene chimeric ventral hippocampus (vHipp)-nucleus accumbens (nAcc) coculture from mouse, electrophysiology, immunocytochemistry, FM1-43 vesicle fusion, and electron microscopy techniques to examine the pre- and postsynaptic mechanisms of genetic deficits in Nrg1/ErbB signaling-induced glutamatergic dysfunctions. Reduced presynaptic type III Nrg1 expression along vHipp axons decreases the number of glutamate synapses and impairs GluA2 trafficking in the postsynaptic nAcc neurons, resulting in decreased frequency and amplitude of miniature EPSCs (mEPSCs). Reduced expression of axonal type III Nrg1 along vHipp projections also decreases functional synaptic vesicle (SV) clustering and vesicular trafficking to presynaptic vHipp axonal terminals. These findings suggest that Nrg1/ErbB signaling modulate glutamatergic transmission via both pre- and postsynaptic mechanisms.

## Significance Statement

Presynaptic neuregulin 1 (Nrg1) to postsynaptic ErbB signaling contributes to excitatory synapse formation and plasticity, and disturbances in this signaling are thought to contribute to a number of structural and functional endophenotypes associated with schizophrenia. In this study, we uncover a new role for axonal Nrg1 signaling in the formation and functional maturation of glutamatergic, presynaptic specializations. Axonal signaling by Nrg1 adds an additional layer of complexity to the role of these molecules in neuronal development and might provide additional insight into their contribution to the etiology of schizophrenia.

## Introduction

Schizophrenia is a highly heritable neuropsychiatric illness affecting ∼1% of the world’s population. Alterations of glutamate-mediated synaptic neurotransmission, in particular glutamatergic hypofunction, are implicated in the pathophysiology of schizophrenia, although there is no consensus on either the origin of these dysfunctions or to what extent glutamatergic dysfunctions are causative for the disorder (Paz et al., 2008; Gaspar et al., 2009; Coyle et al., 2012; Lin et al., 2012). The major mechanisms leading to altered glutamatergic synaptic strength include modulating neurotransmitter (glutamate) release at presynaptic terminals and altering the glutamate receptor responses at postsynaptic terminals (González-Forero et al., 2012; Allam et al., 2015).

Studies of candidate schizophrenia risk genes and postmortem analyses have implicated changes in neuregulin 1 (Nrg1) signaling as a contributor to disease-associated endophenotypes (Stefansson et al., 2002; Banerjee et al., 2010). The Nrg1 gene encodes a family of ligands for ErbB receptor tyrosine kinases (Hobbs et al., 2002; Grossmann et al., 2009). Nrg1/ErbB signaling plays a complex role in regulating glutamatergic synaptic transmission. For example, Nrg1/ErbB signaling affects excitatory synaptogenesis on cortical interneurons (Ting et al., 2011) interferes with LTP at hippocampal synapses (Kwon et al., 2005) and is necessary for plasticity at cortical-amygdala synapses (Jiang et al., 2013). A critical question that emerges from these studies is whether altered Nrg1 signaling contributes to the glutamatergic hypofunction seen in patients with schizophrenia and/or psychoses and, if so, how.

Mechanistically, Nrg1 signaling has been shown to alter NMDA (Gu et al., 2005; Hahn et al., 2006; Pitcher et al., 2011) and AMPA receptor levels and trafficking (Abe et al., 2011; Fenster et al., 2012), GABA-A (Okada and Corfas, 2004; Fazzari et al., 2010), nicotinic acetylcholine receptor (nAChRs) (Liu et al., 2001; Kim et al., 2013) expression, and presynaptic targeting of nAChRs (Hancock et al., 2008). These recent studies converge on the idea that critical balance of Nrg1/ErbB signaling is required for maintaining optimal glutamatergic synaptic plasticity.

Glutamatergic projections from the ventral subiculum of the hippocampus innervate the GABAergic medium spiny neurons (MSNs) in the ventral striatum/nucleus accumbens (nAcc). Type III Nrg1 is expressed in the ventral subiculum of the hippocampus but not in ventral striatum; in contrast, the Nrg1 receptor, ErbB4, is widely expressed in the early postnatal ventral striatum (Chen et al., 2008). Recently, it has been demonstrated that bidirectional signaling by the type III isoforms of Nrg1 is essential for establishing and maintaining functional levels of presynaptic α7-containing nAChRs on ventral subicular and cortical glutamatergic projections (Zhong et al., 2008; Jiang et al., 2013). As a result, type III Nrg1 heterozygotes have pronounced deficits in α7 nAChR-dependent presynaptic modulation of glutamate release and compromised glutamatergic synaptic plasticity. In this report, we investigated a more general role for ventral hippocampal presynaptic type III Nrg1 signaling in establishing glutamatergic synapses on nAcc MSNs.

## Materials and Methods

### Ventral hippocampus (vHipp) microslices culture and vHipp-nAcc synaptic coculture

All animal experiments were conducted in accordance with the National Institutes of Health Guide for the Care and Use of Laboratory Animals (NIH Publications No. 80-23, revised 2012). Animals used in these studies were derived from crosses of heterozygous male and female Nrg1^tm1.1Lwr^ mice, in which the exon unique to type III Nrg1 isoform has been disrupted (Wolpowitz et al., 2000; on a C57Bl6/J background).

**Figure 1.**
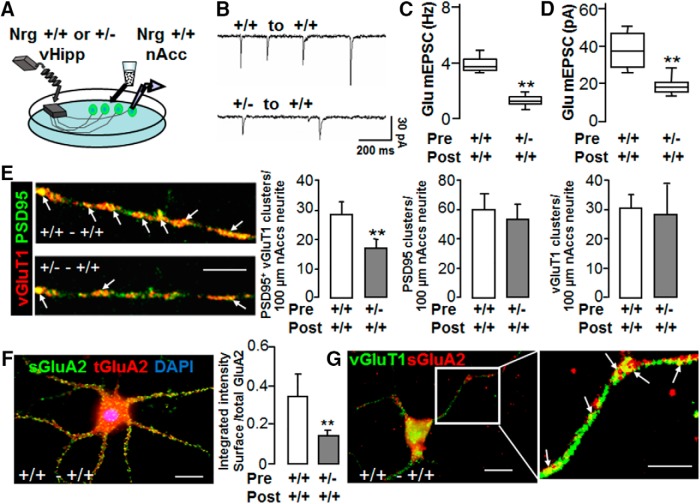
Reduced presynaptic type III Nrg1 signaling impairs glutamatergic synaptic transmission at vHipp-nAcc synapses. ***A***, Schematic of genotype-specific *in vitro* circuits. Glutamatergic transmission at vHipp-nAcc synapses was examined in gene chimeric cocultures. Experiments show ventral hippocampus/subiculum slices from an individual Nrg+/+ or Nrg+/− mouse plated as micro thinned explants. Dispersed nucleus accumbens neurons from WT mice were added 24 h later. ***B***, Representative traces of spontaneous glutamate-receptor mediated synaptic activity (Glu mEPSCs: bicuculline and TTX resistant; CNQX-APV sensitive) recorded from +/+ vHipp to +/+ nAcc synapses (top) and from +/− vHipp to +/+ nAcc synapses (bottom). ***C***, Box plots of mEPSC frequency data from +/+ vHipp to +/+ nAcc and from +/− vHipp to +/+ nAcc reveal more than a 3-fold difference (**p < 0.01, *n* = 8 for each condition) at gene chimeric synapses compared with WT- WT synapses. ***D***, Box plots of mEPSC amplitude data from +/+ vHipp to +/+ nAcc and from +/− vHipp to +/+ nAcc reveal a 2-fold difference (**p < 0.01, *n* = 8 for each condition) at gene chimeric synapses compared with WT- WT synapses. ***E***, Examination of pre and postsynaptic markers in gene chimeric vHipp-nAcc cocultures. After 5-7 d *in vitro*, cocultures were fixed, permeabilized, and stained with antibodies targeted to vesicular glutamate transporter 1 (vGluT1; red) and to PSD95 (green). Red “clusters” of vGluT1 are colocalized with PSD95 (green) on neurites of dispersed nAcc neurons innervated by +/+ vHipp (***E***, top left) or +/− vHipp (***E***, bottom left; scale bar, 10μm). The arrows indicate colocalization of PSD95 with vGLuT1 (yellow puncta) along neurites of nAcc MSNs. The number of PSD95 positive/vGluT1 clusters along neurites of dispersed nAcc neurons innervated by +/+ vHipp were significantly greater than those innervated by +/− vHipp inputs (28 ± 4 per 100 μm, *n* = 9, 5 vs 17 ± 3 per 100 μm *n* = 6, 3; where n= the number of samples/experiment and the number of separate experiments; **p < 0.01; ***E***, middle, left). The number of PSD95 clusters along neurites of nAcc neurons were counted and the bar graph (***E***, middle, right) showed no difference between +/+ and +/− vHipp innervation (+/+vHipp to +/+nAcc: 60 ± 10, *n* = 9, 5 vs +/−vHipp to +/+nAcc: 54 ± 10, *n* = 6, 3; *p* > 0.05). The number of vGluT1 clusters along neurites of nAcc neurons were also counted and the bar graph (***E***, right) showed no difference between +/+ and +/− vHipp innervation (+/+ vHipp to +/+ nAcc: 31 ± 4, *n* = 9, 5 vs +/− vHipp to +/+ nAcc: 29 ± 8, *n* = 6, 3; *p* > 0.05). ***F***, Examination of surface versus total glutamate A2/A3 receptor subtypes (GluA2) in gene chimeric vHipp-nAcc cocultures. After 5-7 d *in vitro*, cocultures were stained with antibodies targeted to GluA2. For labeling of sGluA2 (green), the cultures were incubated with anti-GluR2 antibody, extracellular, for 45 min before fixation, and then, cultures were fixed, permeabilized, and total GluA2 (red) were recognized with anti-GluR2 + GluR3 antibody, C terminal. Clusters of surface (green) vs total GluA2/3 (red) can be found on neurites of dispersed nAcc neurons innervated by vHipp axons (***F***, left panel**;** scale bar, 5 μm). The ratio of integrated intensities of sGluA2 versus total GluA2 between gene chimeric conditions differed in a statistically significant manner (+/+ vHipp to +/+ nAcc: 0.36 ± 0.07, *n* = 10, 4 vs +/− vHipp to +/+nAcc: 0.18 ± 0.02, *n* = 8, 3; **p < 0.01; ***F***, right-hand panel). ***G***, Examination of synapse formation of sGluA2 (red) and vGluT1 (green) along nAcc neurites in vHipp-nAcc cocultures. After 5-7 d *in vitro*, cocultures were incubated with anti-GluR2 antibody recognizing an extracellular epitope, for 45 min to label sGluA2, and then, cultures were fixed, permeabilized, and stained with antibody targeted to vGluT1. Representative micrographs of vGluT1 and sGluA2 colocalization are shown, the arrows are examples of those colocalized sites (yellow puncta) of sGluA2 (red) and vGLuT1 (green) along neurites of nAcc MSNs (***G***; scale bar, 5 μm).

**Figure 2.**
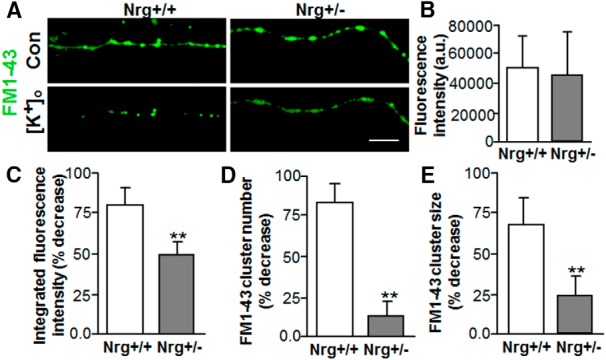
Reduced presynaptic type III Nrg1 signaling decreases depolarization-induced FM1-43 destaining along vHipp axons. Cultures of vHipp microslices from WT (Nrg+/+) or heterozygous (Nrg+/−) mice were loaded with FM1-43. Representative micrographs of WT (Nrg+/+; ***A***, left) and Nrg+/− (***A***, right) vHipp axons (loaded with FM1-43, green) before (***A***, top) and after (***A***, bottom) depolarization with elevated extracellular K^+^ are shown. Scale bar, 10μm. ***B***, Bar graph showed no difference in the initial FM1-43 fluorescence intensities after K^+^-dependent loading between +/+ and +/− vHipp axons. The efficacy of depolarization in eliciting release was assayed from determinations of FM1-43 staining including measures of the % decrease with depolarization as well as any differences in the number and/or size of the FM1-43 clusters (***C--E***). ***C***, The overall FM1-43 fluorescence intensity decreased along vHipp axons after depolarization in both Nrg+/+ and Nrg+/− vHipp axons, the magnitude of the effect is significantly lower for the Nrg+/− vHipp axons compared with control whether assessed as the percentage of total fluorescence intensity (***C***) or the percentage decrease in cluster number (***D***) or in the size of the FM1-43 clusters (***E***). Bar graphs of mean ± SEM of eight independent experiments. The effect of depolarization on transmitter release (assayed as FM1-43 destaining) significantly depressed in the Nrg+/− vHipp compared with the Nrg+/+ controls (**p < 0.01).

**Figure 3.**
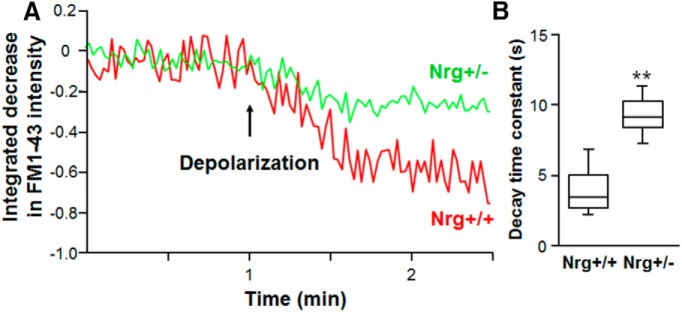
Reduced presynaptic type III Nrg1 slows depolarization-induced FM1-43 release. ***A***, Spinning disk confocal images of FM1-43-loaded axons from either +/+ or +/− vHipp were collected every 1.5 s for 5 min, and FM1-43 fluorescence intensities were calculated and quantified as a normalized integrated intensity at each time point. The percentage decrease of normalized integrated intensity at an individual 1-µm spot (3 pixels) along vHipp axons were plotted versus time. Representative plots of the release time course from Nrg+/+ (red) and Nrg+/− (green) vHipp axon are shown before and after depolarization. ***B***, Box plot of pooled data shows slower decay time constant (τ) of high K^+^ depolarization-induced FM1-43 destaining along axons from Nrg+/− (∼8.5 s, *n* = 20, 8) compared with Nrg+/+ (∼4.8 s, *n* = 36, 10 experiments; **p < 0.01).

**Figure 4.**
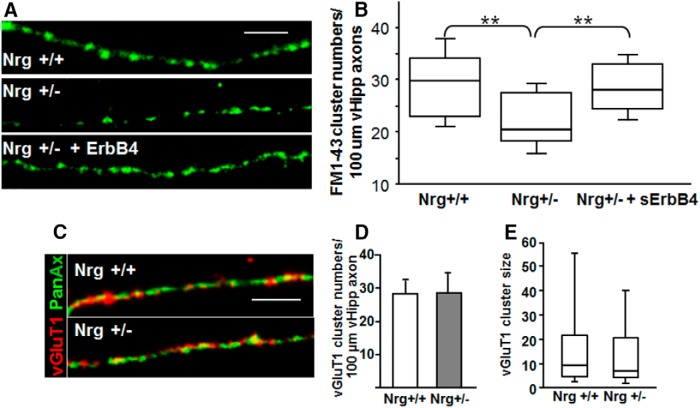
ErbB4-induced back-signaling rescues presynaptic phenotype of type III Nrg+/− vHipp axons. ***A***, Representative micrographs of Nrg+/+ (top), Nrg+/− (middle) and Nrg+/− with 24 h of treatment of soluble ErbB4 (bottom) vHipp axons loaded with FM1-43 (green) to visualize sites of vesicle clusters (scale bar, 10μm). ***B***, Box plot of pooled data shows a significant decrease in the number of FM1-43-stained clusters along axons from Nrg+/− compared with Nrg+/+ vhipp axons. Following 24 h of treatment of Nrg+/− axons with 2 ng/ml soluble ErbB4-ECD, the number of FM1-43-stained clusters along Nrg+/− axons was rescued to Nrg +/+ levels; **p < 0.01. ***C***, Representative micrographs of Nrg+/+ (top), Nrg+/− (bottom) vHipp axons stained with antibodies recognizing vGluT1 (red), and a pan axonal marker (green) are shown. Scale bar, 10μm. ***D***, Quantification of vGluT1 cluster numbers along vHipp axons from Nrg+/+ (28 ± 4 per 100 μm) or Nrg+/− (28.5 ± 5.5 per 100 μm) shows that there is no significant difference between conditions. ***E***, Box plot of pooled data shows no significant difference in the size of vGluT1 clusters along axons from Nrg+/− compared with Nrg+/+.

**Figure 5.**
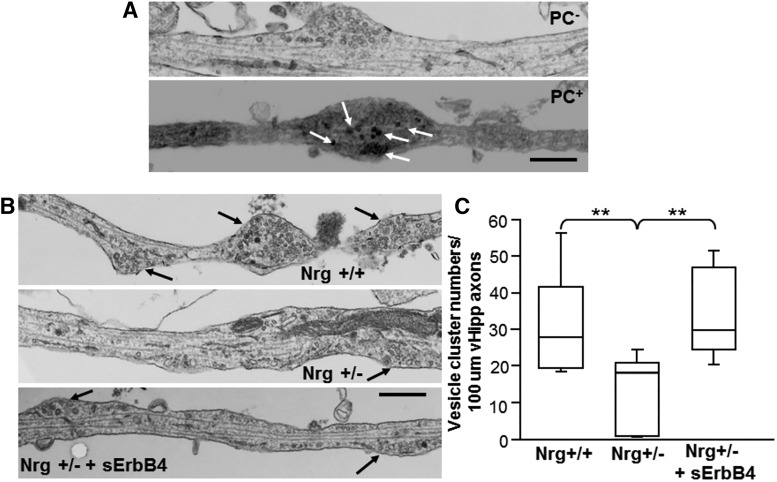
ErbB4-induced back-signaling reverses ultrastructural defects in the number of SV clusters. ***A***, Representative electron micrographs of control (nonphotoconverted, PC^-^, top) and photoconverted (PC^+^; bottom) SVs along vHipp axons are shown (scale bar, 500 nm). The arrows indicate electron-dense material resulting from photoconversion of FM1-43 dye that is confined to the lumen of photoconverted s SVs. ***B***, Representative electron micrographs of Nrg+/+ (top), Nrg+/− (middle), and Nrg+/− after 24 h of treatment with soluble ErbB4 (bottom) vHipp axons are shown. The arrows indicate the location of vesicle clusters (defined as ≥15 vesicles within less than a vesicle diameter of one another; scale bar, 500 nm). ***C***, Box plot of pooled data (>40 axons from 2 separate experiments) shows a significant decrease in the number of vesicle clusters along axons from Nrg+/− compared with Nrg+/+; **p < 0.01. Following 24 h of treatment with 2-ng/ml soluble ErbB4-ECD, the total number of vesicle clusters per 100-µm axon length along Nrg+/− vHipp axons was rescued to levels comparable to Nrg+/+ control (**p < 0.01).

For vHipp microslices culture, ventral CA1 and subiculum area (vHipp) from WT (+/+) or Nrg1^tm1.1Lwr^ heterozygous (+/−) mice (postnatal d 0-3, P0–P3) were dissected, sliced into 150 × 150 μm pieces, and then plated onto poly-D-lysine/laminin-coated glass coverslips (BD Sciences) in a minimal volume (50 μl) of culture media [Neurobasal, 2% B-27 (GIBCO) and 20 ng/ml of brain-derived neurotrophic factor (R&D Systems)]. After the microslices attached to the substrate, 100 μl of additional culture media was added. Prior studies using a similar vHipp microsclice preparation in the absence of postsynaptic targets demonstrated that nicotine regulated calcium signaling and FM1-43 vesicle cycling in glutamatergic axons from the vHipp microslices (Zhong et al., 2008; Zhong et al., 2013; Zhong et al., 2015). Here, we use vHipp-nAcc synaptic cocultures to examine the effects of Nrg1/ErbB4 signaling on functional glutamatergic synapse formation (Fig. 1), and we use vHipp microslices cultured alone to focus on axonal presynaptic-like specializations (Figs. 2-5).

For vHipp-nAcc synaptic cocultures, nAccs (ED18-P1) from WT mice (C57BL/6J) were dissected out and dispersed with 0.25% trypsin (GIBCO) for 15 min at 37°C, followed by gentle trituration in culture media. Dispersed nAcc neurons were added to the vHipp microslices plated the prior day at 0.25 ml/coverslip. All cultures were maintained in a humidified 37°C, 5% CO_2_ incubator.

### Electrophysiological recordings

Synaptic currents were recorded from WT nAcc neurons contacted with vHipp axons from WT or Nrg1 +/− mice by whole-cell configuration of the patch clamp technique. Briefly, after 5-7 d *in vitro*, vHipp-nAcc cocultures in a recording chamber were continuously superfused with extracellular solution: 145 mM NaCl, 3 mM KCl, 2.5 mM CaCl_2_, 10 mM HEPES, and 10 mM glucose, pH 7.4. The intracellular solution included: 3 mM NaCl, 150 mM KCl, 1 mM MgCl_2_, 1 mM EGTA, 10 mM HEPES, 5 mM MgATP, and 0.3 mM NaGTP, pH 7.2. Bicuculline (Tocris) and TTX (Sigma) were included in the superfusion as noted. Synaptic currents were filtered at 10 kHz with an 8-pole Bessel filter (DC Amplifier/Filter, Warner Instruments) before acquisition and digitization through a DigiData 1200B A/D interface with pCLAMP 8 (Molecular Devices). The amplitudes and frequency of TTX resistant, spontaneous synaptic currents were measured with MiniAnalysis (RRID: SCR_014441 Synaptosoft).

### Immunocytochemistry and reagents

After 5-7 d *in vitro*, vHipp-nAcc cocultures were fixed in 4% paraformaldehyde/4% sucrose/PBS (20 min, room temperature, (RT)), permeabilized with 0.25% Triton X-100/PBS (5 min, RT), blocked with 10% normal donkey serum in PBS (30 min, RT), and then incubated in primary antibodies overnight at 4°C. The following primary antibodies were used: anti-PSD95 (1:500, catalog #P246 RRID: AB_260911; Sigma-Aldrich), anti-vesicular glutamate transporter 1 (1:250, catalog #135 302; Synaptic Systems), anti-pan-axonal neurofilament marker (1:1000, Santa Cruz Biotechnology), anti-GluA2, extracellular, clone 6C4 (1:500, catalog #MAB397; EMD Millipore), and anti-GluA2 + GluA3, C-terminal (1:500, catalog #PA1-4660; Thermo Fisher Scientific). Cultures were washed and incubated in secondary antibodies conjugated to Alexa Fluor 488 (1:500, catalog #A-21206 RRID: AB_141708; Invitrogen) or Alexa Fluor 594 (1:500, catalog #A-21203 RRID: AB_141633; Invitrogen) for 1 h at RT. Slips were mounted using VectaShield (with DAPI, catalog #H-1200 RRID: AB_2336790; Vector Laboratories), and images were captured using a microscope (Axio Imager A1; Carl Zeiss) equipped with Plan-Apochromat objectives (63× oil with 1.4 NA), a CCD camera (Hamamatsu), and MetaMorph software (version 7.1, MetaMorph Microscopy Automation and Image Analysis Software, RRID:SCR_002368; Molecular Devices).

To label surface GluA2 (sGluA2), vHipp-nAcc cocultures were incubated in anti-GluA2 antibody, extracellular, clone 6C4 (1:500) for 45 min at 37°C before fixation. Then, total GluA2 was visualized with anti-GluA2 + GluA3 antibody, C-terminal (1:500) with the normal staining procedures.

Morphologic synaptic contacts were identified as puncta of presynaptic (vGluT1) and postsynaptic (PSD95, sGluA2) marker proteins that are formed during synapse formation and maturation (Ippolito and Eroglu, 2010). First, the number of vGluT1 and PSD95 clusters were measured separately along nAcc neurites contacted by vGluT1-positive vHipp axons using MetaMorph software. The number of PSD95-positive vGluT1 clusters (colocalization) along nAcc neurites were counted as synaptic contacts. We considered vGluT1 and PSD95 (or sGluA2) puncta to be colocalized if markers directly overlapped (yellow puncta) or were closely apposed to each other (Chao et al., 2007). The lengths of vHipp axonal projections and nAcc neurites were also measured in MetaMorph. The overall ratio of sGluA2 to total GluA2 from all neurites was also quantified.

### FM1-43-based imaging and analysis

After 5-7 d *in vitro*, vHipp microslices cultures were maintained in an imaging chamber (Live Imaging Services; containing 1 ml of fresh normal HBS) mounted on a Olympus IX81 DSU (spinning disk confocal) microscope (Olympus America) under continuous superfusion (1 ml/min) with HBS containing 2 μM tetrodotoxin (TTX; Tocris), 10 μM bicuculline (Tocris), 50 μM D-AP-5 (Tocris), and 20 μM CNQX (Tocris). vHipp microslices were loaded with 10 μM FM1-43 (Invitrogen) in 56 mM K^+^ ACSF for 90 s, external dye was washed away in Ca^2+^-free HEPES-buffered saline (HBS, 135 mM NaCl, 5 mM KCl, 1 mM MgCl_2_, 10 mM HEPES, and 10 mM glucose, pH 7.4) containing ADVASEP-7 (0.1 mM; Sigma) to scavenge membrane-bound FM1-43 for 15 min. Fluorescence images of vHipp axons were collected by a Plan-Apochromat objective (60× oil with 1.4 NA, excitation 488 nm, emission 530 nm) and captured with a CCD camera (Hamamatsu) every 1.5 s for 5 min. Image acquisition was performed using Slidebook software (version 5; Olympus). After 1 min of baseline data collection, 56 mM K^+^ ACSF without FM1-43 was applied for 120 s to confirm that high K^+^ depolarization can induce destaining of FM1-43 dye–filled vesicles. The total amount of releasable fluorescence at each synaptic bouton was calculated from the difference between fluorescence intensity after staining (and before destaining) and after depolarization induced destaining (Δ*F* = *F*_staining_-*F*_destaining_). The fraction of fluorescence intensity decrease after depolarization was calculated as (F_decrease_%=Δ*F/F*_staining_). The number and size of FM1-43-positive puncta before and after high K^+^ depolarization were measured and compared along vHipp axons from Nrg1^tm1.1Lwr^ wild type (+/+) vs heterozygous (+/−) mice using MetaMorph software. The lengths of axonal projections were also measured by tracing vHipp projections in MetaMorph.

For analyzing the time course of high K^+^ depolarization-induced FM1-43 destaining, all frames of the raw FM1-43 fluorescence images were saved as Slidebook files and then exported as a series of TIF format images that were then imported to MetaMorph software and transferred as Z-stack images for further analyses. After setting the threshold of the FM1-43 fluorescence, the integrated intensity of the FM1-43 signals along vHipp axons before and after high K^+^ depolarization was calculated. FM1-43 fluorescence data are displayed as a normalized integrated intensity: [Δ*F/F_0_ = (F - F_0_)/F_0_*], where *F_0_* is the background-corrected prehigh K^+^ depolarization FM1-43 fluorescence. Data were analyzed further using Excel software.

### Electron microscopy

After 5-7 d *in vitro*, vHipp microslices grown on Thermanox plastic coverslips (NUNC/Electron Microscopy Sciences) were fixed for 30 min in a mixture of cold 2% paraformaldehyde and 2% glutaraldehyde in 0.1 M phosphate buffer (PB), pH 7.4. After several washes in PB, explants were postfixed with 2% osmium tetroxide for 30 min, *en bloc* stained with aqueous 1% uranyl acetate for 30 min, dehydrated through an ascending series of ethanols, followed by acetonitrile, and embedded in Embed 812 resin (Electron Microscopy Sciences) for 48 h at 60°C. Ultrathin sections (60-90 nm) were cut and then stained with 1% methanolic uranyl acetate and 0.3% aqueous lead citrate, and observed with a JEOL 1200EX transmission electron microscope.

### Photoconversion of FM1-43

The photoconversion procedure was modified from a prior study (Harata et al., 2001). After FM1-43 staining, cultures on glass coverslips containing vHipp axons were fixed with 2% glutaraldehyde in 100 mM PBS for 20 min, and washed with glycine (100 mM in PBS) for 1 h, and then washed in ammonium chloride (100 mM in distilled water) for 5 min. After brief rinsing in PBS, axons were incubated in DAB (1 mg/ml in PBS) for 20 min. Fluorescence excitation light was then continuously applied for 10-20 min in DAB solution. Cultures were then washed in ice-cold PBS and processed for EM.

After DAB photoconversion, microslices cultures were briefly rinsed with PBS and then postfixed with 1% osmium tetroxide containing 0.8% potassium ferricyanide for 30 min. The coverslips were *en bloc* stained with aqueous 2% uranyl acetate for 20 min, dehydrated through an ascending series of ethanols, and embedded in Durcupan resin for 48 h at 60°C.

Glass coverslips were removed by submerging in liquid nitrogen. Ultrathin sections (60-90 nm) were cut and then stained with 1% methanolic uranyl acetate and 0.3% aqueous lead citrate and observed with a JEOL 1200EX transmission electron microscope.

### Statistical analysis

All data were expressed as mean ± SEM unless otherwise indicated and analyzed with StatView (SAS Institute) or Microsoft Excel (Microsoft) software. Superscript letters listed with *p* values correspond to the statistical tests shown in Table 1.

**Table 1.** Statistical analyses used in this study

Line	Data structure	Type of test	*p*
a	Normal distribution	Two-sample Kolmagorov–Smirnov test	0.005
b	Normal distribution	Two-sample Kolmagorov–Smirnov test	0.008
c	Normal distribution	Student’s *t* test	0.001
d	Normal distribution	Student’s *t* test	0.45
e	Normal distribution	Student’s *t* test	0.08
f	Normal distribution	Student’s *t* test	0.003
g	Normal distribution	Student’s *t* test	0.35
h	Normal distribution	Student’s *t* test	0.005
i	Normal distribution	Student’s *t* test	0.001
j	Normal distribution	Student’s *t* test	0.001
k	Normal distribution	Student’s *t* test	0.001
l	Normal distribution	One-way ANOVA tests	0.008
m	Normal distribution	One-way ANOVA tests	0.005
n	Normal distribution	Student’s *t* test	0.56
o	Normal distribution	Student’s *t* test	0.58
p	Normal distribution	One-way ANOVA tests	0.001
q	Normal distribution	One-way ANOVA tests	0.001

## Results

We employed a gene chimeric coculture system to examine whether and how alteration of presynaptic type III Nrg1 signaling modulates glutamatergic transmission. The ventral hippocampus (vHipp) microslices extend glutamatergic axons that synapse on dispersed MSNs from nAcc (Fig. 1*A*). Postsynaptic currents were recorded from nAcc dispersed neurons innervated by either WT (+/+) or type III Nrg1 heterozygous (+/−) vHipp. First, we tested the spontaneous glutamate-receptor mediated synaptic activity, i.e., miniature EPSCs (Glu mEPSCs: bicuculline and TTX resistant; CNQX-APV sensitive), in nAcc MSNs from either WT vHipp (+/+) to WT nAcc (+/+) (Fig. 1*B*, top) or Het vHipp (+/−) to WT nAcc (+/+) (Fig. 1*B*, bottom) cocultures. Both the Glu mEPSC frequency (∼4 Hz vs 1.5 Hz; Fig. 1*C*; *n* = 8 WT to WT versus *n* = 8 Het to WT; *p* = 0.005^a^) and amplitude (∼35 pA vs 18 pA; Fig. 1*D*, *p* = 0.008^b^) were significantly reduced when measured in WT MSNs receiving type III Nrg1 heterozygote vHipp input compared with WT input. These data, along with other published results, demonstrate that the level of presynaptic type III Nrg1 affects glutamatergic synaptic transmission (Zhong et al., 2008; Jiang et al., 2013).

The observed decrease in mEPSC frequency could reflect either a decrease in the total number of synapses or/and a decrease in the probability of spontaneous glutamatergic synaptic vesicle (SV) release. To determine whether reduced presynaptic type III Nrg1 expression altered the number of glutamatergic synapses formed on WT MSNs, vHipp-nAcc cocultures were fixed, permeabilized, and stained with antibodies recognizing the presynaptic marker, vGluT1, and the postsynaptic marker, PSD95. Clusters of vGluT1 (Fig. 1*E*, red) were colocalized with the postsynaptic marker PSD95 (green) along neurites of dispersed nAcc MSNs innervated by +/+ (Fig. 1*E*, left top) or +/− (Fig. 1*E*, left bottom) vHipp axons. The number of PSD95^+^/vGluT1^+^ clusters along nAcc MSNs neurites was significantly decreased (Fig. 1*E*, middle left) when the WT nAcc MSNs were innervated by type III Nrg1 Het (+/−) vHipp axons (17 ± 3 per 100 μm of nAcc neurites) compared with WT nAcc MSNs innervated by Nrg1 WT (+/+) vHipp axons (28 ± 4 per 100 μm of nAcc neurites; *p* = 0.001^c^). There was no significant difference (Fig. 1*E*, right; *p* = 0.45^d^) in the number of vGluT1 cluster numbers along nAcc neurites when innervated by WT vHipp axons (31 ± 4 per 100 μm of nAcc neurites) compared with Nrg+/− vHipp axons (29 ± 8 per 100 μm of nAcc neurites). The PSD95 cluster numbers along nAcc neurites innervated by Nrg+/− vHipp axons (54 ± 10 per 100 μm of nAcc neurites) compare to innervated by Nrg+/+ vHipp axons (60 ± 10 per 100 μm of nAcc neurites) showed a slight, but not significant, decrease (Fig. 1*E*, middle right; *p* = 0.08^e^). This indicates that reduced presynaptic type III Nrg1 expression decreased the numbers of synaptic contacts formed on Nrg1 WT postsynaptic MSNs in these vHipp-nAcc cocultures. Neither the frequency nor the amplitude of TTX-resistant inhibitory postsynaptic currents (mIPSCs) were altered by the Nrg1 genotype of the vHipp explants (data not shown), indicating that formation of GABAergic synapses between nAcc MSNs was not affected by type III Nrg1 on the vHipp glutamatergic inputs.

The decreased amplitude of individual mEPSCs could result from a decrease in the number of postsynaptic glutamate receptors (most likely AMPA-type receptors under these recording conditions). To determine whether alterations of presynaptic type III Nrg1 signaling affected the numbers of postsynaptic AMPARs, we quantified the ratio of surface to total GluA2-containing AMPARs on WT nAcc MSNs innervated by either WT vHipp or type III Nrg1 Het vHipp explants. Live cocultures were stained with a GluA2 antibody that recognizes extracellular epitopes, after which the cocultures were fixed, permeabilized and stained with a different GluA2 antibody. Clusters of sGluA2 (green) and total GluA2 (red) were present on both soma and neurites of WT nAcc MSNs innervated by vHipp axons (Fig. 1*F*). The overall ratio of sGluA2 to total GluA2 integrated fluorescence intensity along neurites of dispersed nAcc neurons was significantly decreased (Fig. 1*F*, right; *p* = 0.003^f^) when the neurons were innervated by type III Nrg1 Het (+/−) vHipp axons (0.18 ± 0.02) compared with WT nAcc MSNs innervated by WT Nrg1 (+/+) vHipp axons (0.36 ± 0.07). The ratio of surface to total GluA2 quantified over MSNs soma was not as affected by presynaptic type III Nrg1 genotype (WT ∼4.5 vs Het ∼3.5; data not shown).

To determine whether the sGluA2 staining along nAcc neurites were at synapses, we stained cocultures of nAcc neurons innervated by WT vHipp axons for sGluA2 and vGluT1 (Fig. 1*G*). The majority of sGluA2 staining along the neurites was colocalized with vGluT1 clusters indicating that many of these clusters were likely to be sites of synaptic contacts (Fig. 1*G*).

Taken together, these data demonstrate that reduction in the levels of presynaptic type III Nrg1 results in a 40-60% decrease in excitatory synapse formation on WT nAcc MSNs and that the level of postsynaptic receptors present on the synapses that do form is decreased as well.

Formation of glutamatergic synapses onto striatal MSNs requires glutamatergic transmission (Kozorovitskiy et al., 2012). Given that it has been demonstrated that presynaptic type III Nrg1 back-signaling is critical for targeting various receptors to presynaptic sites (Hancock et al., 2008; Zhong et al., 2008; Canetta et al., 2011; Hancock et al., 2011), we next asked whether alterations of presynaptic type III Nrg1 signaling could affect neurotransmitter release. To focus specifically on the effect of type III Nrg1 signaling in vHipp axons, the next series of experiments used vHipp microslices that were cultured in the absence of postsynaptic nAcc MSNs. vHipp microslices from either WT or type III Nrg1 Het mice were loaded with FM1-43 and then depolarization-induced destaining of the readily releasable pool of vesicles (RRP) was quantified by live cell imaging. FM1-43-labeled vesicle puncta in both WT (+/+) (Fig. 2*A*, left top) and type III Nrg1 Het (+/−) (Fig. 2*A*, right top) vHipp axons showed no differences in the initial FM1-43 fluorescence intensities after K^+^-dependent loading (Fig. 2*B*; *p* = 0.35^g^). Also, there are no significant changes in FM1-43 fluorescence intensities and cluster numbers for at least 30 min in the absence of stimulation, indicating that there was minimal photo-bleaching under our imaging conditions (data not shown). After high K^+^ (56 mM) ACSF application, the fluorescence intensity of all puncta along WT (+/+) vHipp axons (Fig. 2*A*, left bottom, *B*) decreased by ∼80%, reflecting depolarization-induced exocytosis of dye from SVs. In contrast, the decrease in depolarization-induced fluorescence intensity of FM1-43 puncta from type III Nrg1 Het (+/−) axons was significantly reduced compared with WT (Fig. 2*A*, right bottom, *C*; ∼50% reduction in fluorescence, *p* = 0.005^h^). Over 80% of FM1-43-loaded vesicle clusters along WT vHipp axons were totally destained by depolarization (Fig. 2*D*) and the size of the partially destained clusters decreased by 70% (Fig. 2*E*). In contrast only ∼20% of FM1-43 clusters totally destained along type III Nrg1 Het (+/−) axons (Fig. 2*D*; WT vs Het, *p* = 0.001^i^), and there was only about a 25% decrease in overall cluster size (Fig 2*E*; WT vs Het, *p* = 0.001^j^).

We also quantified the time course of depolarization-induced FM1-43 release from individual synaptic vesicular clusters. Images of FM1-43 along vHipp axons were recorded every 1.5 s for 5 min using live spinning disk confocal microscopy, and changes in FM1-43 fluorescence intensities were used to calculate decay time constants (Fig. 3*B*). The fraction of normalized integrated intensity decrease at single clusters (three pixels ∼ 1 µm) along vHipp axons were plotted versus time (Fig. 3*A*). Following depolarization, WT axons destained with a time constant of ∼4.8 s, whereas the type III Nrg1 Het (+/−) axons destained at a significantly slower rate and as noted above, failed to fully destain (Fig. 3*B*; time constant ∼8.5 s; WT vs Het, *p* = 0.001^k^). Based on these measures, it appears that presynaptic type III Nrg1 contributes to the normal pattern and rate of SV cycling and neurotransmitter release along vHipp axons; the majority of the loaded vesicles in the type III Nrg1 Het axons either failed to destain, or partially destained at a slower rate, on subsequent depolarizations.

In addition to overall alterations in vesicle cycling in type III Nrg1 Het vHipp axons, the total number of FM1-43-labeled clusters was reduced compared with Nrg1 WT axons (Fig. 4*A*,*B*). In contrast, the number (*P* = 0.56^n^) and size (*P* = 0.58^°^) of vGluT1-containing clusters recognized by antibody staining did not differ by genotype (Fig. 4*C--E*). vGluT1 cluster number (Fig. 4*D*) along vHipp axons from Nrg1 WT (+/+) (28.1 ± 4.2 per 100 μm) were comparable to axons from type III Nrg1 Hets (+/−) (28.5 ± 5.5 per 100 μm). After loading with FM1-43, we quantified the numbers of vesicle clusters (puncta) in WT (Fig. 4*A*, top) and type III Nrg1 Het (+/−) (Fig. 4*A*, middle) vHipp axons. Analyses of pooled data (Fig. 4*B*) show a significant decrease in the number of FM1-43-stained clusters along axons from type III Nrg1 Het (+/−) (22 ± 2 per 100 μm of vHipp axon) compared with WT (+/+) (29 ± 1 per 100 μm of vHipp axon, *p* = 0.008^l^); there was no statistically significant difference in the size of FM1-43-positive clusters between the genotypes (data not shown, but see Figure 5 below). To confirm that presynaptic type III Nrg1 back-signaling contributes to the regulation of SV clustering, we stimulated type III Nrg1 back-signaling in type III Nrg1 Het cultures by adding soluble ErbB4 extracellular domain to the media. After treatment with soluble ErbB4-ECD for 24 h, microslices were loaded with FM1-43 (Fig. 4*A*, bottom). Stimulation of type III Nrg1 back-signaling in vHipp axons from type III Nrg1 Het animals restored SV cluster numbers to wild type levels (28 ± 1 per 100 μm of vHipp axon; Fig. 4*B*; *p* = 0.005^m^).

To investigate the effect of reducing presynaptic type III Nrg1 signaling on the ultrastructural organization of SVs, we used transmission electron microscopic examination of ultrathin sections of vHipp microslices cultures with extensive axonal arbors. Varicosities containing clusters of apparent SVs were clearly seen along WT axons (Fig. 5*A*,*B*). Following FM1-43 loading and photoconversion, (Harata et al., 2001), we found electron-dense materials resulting from photoconversion of FM1-43 dye were essentially confined to the lumen of photoconverted (PC^+^) SVs (Fig. 5*A*, bottom), whereas non-photoconverted (PC^-^) SVs have a typical translucent appearance (Fig. 5*A*, top), indicating that FM1-43-labeled puncta correspond to vesicle clusters visualized by electron microscopy.

Although clusters of 20-60, 30 nm vesicles were seen along axons from both WT (+/+) and type III Nrg1 Het (+/−) vHipp microslices (Fig. 5*B*, top middle), there were fewer clusters per 100 µm of type III Nrg1 Het axon compared with the Nrg1 WTs (Fig. 5*C*): 13 ± 3 per 100 μm of type III Nrg1 Het vHipp axon compared with 31 ± 4 per 100 μm of WT vHipp axon (*P* = 0.001^p^). The organization of vesicles into clusters along the axon was under the control of type III Nrg1 back-signaling, a 24-h treatment of type III Nrg1 Het (+/−) microslices cultures with soluble ErbB4-ECD, increased the number of vesicle clusters (Fig. 5*B*, bottom, *C*) to WT levels (34 ± 4 per 100 μm of vHipp axon, *p* = 0.001^q^). Taken together, these findings suggest that reduced presynaptic type III Nrg1 signaling impairs the organization of SVs into distinct clusters, and this deficit can be rescued by stimulating type III Nrg1 back-signaling.

## Discussion

We have used a gene chimeric, synaptic coculture between vHipp microslices (presynaptic input) and dispersed nAcc MSNs (as the postsynaptic target) to examine the effect of selective reduction of presynaptic type III Nrg1 expression on glutamatergic transmission. Reduction of presynaptic type III Nrg1 expression had multiple effects on glutamatergic synaptic transmission, including decreases in both amplitude and frequency of MSNs mEPSCs. These changes were associated with decreased morphologic synapse numbers (vGluT1 x PSD95 staining), decreased trafficking of GluA2-containing AMPA receptors to dendritic surfaces and disrupted organization of functional SVs in the vHipp axons.

One major mechanism to modify glutamatergic synaptic transmission is to alter the number of synapses at the glutamatergic terminals (Chao et al., 2007). We used colocalized puncta of the presynaptic and postsynaptic markers, vGluT1 and PSD95 (Melone et al., 2005; Chao et al., 2007; Stevens et al., 2007) to quantify vHipp-nAcc glutamatergic synapse number and found decreases at nAcc MSNs innervated by type III Nrg1 heterozygous vHipp axons. These results are consistent with prior findings that have examined the role of Nrg1/ErbB4 signaling in the establishment of excitatory input to cortical GABAergic interneurons (Chen et al., 2010). Nrg1 activation of ErbB4 on axons of cortical GABAergic interneurons has been shown to enhance activity dependent GABA release (Woo et al., 2007). This provides an additional mechanism by which Nrg1/ErbB4 signaling can modulate the function of GABAergic neurons. Although we did not see changes in mIPSC frequency or amplitude in our gene chimera cocultures, we cannot rule out a similar interaction between axonal type III Nrg1 and MSNs axonal ErbB4. Thus, perturbation of Nrg1/ErbB4 signaling impairs maturation of glutamatergic synaptic input to both GABAergic interneurons and striatal GABAergic projection neurons, possibly contributing to widespread glutamatergic hypofunction (Li et al., 2007).

The decreased mEPSC amplitude recorded at nAcc neurons innervated by type III Nrg1 heterozygous vHipp axons indicates decreased postsynaptic receptor responsiveness. When cocultures were stained with an antibody-recognizing cell sGluA2 containing AMPA receptors, it was clear that reduction of presynaptic type III Nrg1 levels impaired postsynaptic trafficking of sGluA2 receptors to neurites or reduced accumulation of sGluA2 at postsynaptic sites. The decrease in surface to total GluA2 levels was only seen on neurites and not on MSNs soma, indicating that the effect on trafficking is not to the cell surface per se. Previous studies (Gu et al., 2005; Hahn et al., 2006; Li et al., 2007; Abe et al., 2011; Pitcher et al., 2011; Fenster et al., 2012), largely in hippocampus or cortex, have shown that alterations of Nrg1/ErbB4 signaling affect glutamatergic synaptic transmission and plasticity by modulating the expression, internalization and insertion of NMDA and AMPA receptors. Here, we found that genetic defects in vHipp type III Nrg1 signaling impaired AMPA receptor trafficking from intracellular sites to the MSNs surface.

It is likely that the decrease in mEPSC frequency recorded from nAcc MSNs reflects both a decrease in total synapse number as noted above, and a reduction of spontaneous glutamate release at presynaptic terminals. Using both live imaging and high resolution EM, we have demonstrated that presynaptic type III Nrg1 signaling contributes to the formation of functional clusters of SVs and contributes to the recycling of vesicle pools.

Neurotransmitter release involves a cycle of calcium dependent exocytotic fusion of SVs followed by local endocytic recycling (Alabi and Tsien, 2012; Südhof, 2013; Gauthier-Kemper et al., 2015). Physiologically, SVs are distinguished based on their release probability: there is a so-called readily releasable pool (RRP) consisting of vesicles that are released first following depolarization, and there is a reserve pool (RP), which replenishes the RRP on its depletion. Activity-dependent FM1-43 loading and destaining of the RRP has been widely used to examine the property of neurotransmitter release (Tokuoka and Goda, 2008; Blundon et al., 2011; Upreti et al., 2012). Using FM1-43, we found both an absolute decrease in exocytosis of SVs and a decreased rate of release at type III Nrg1 heterozygous presynaptic specializations after depolarization. Given that FM1-43 loading initially required depolarization-induced vesicle fusion followed by endocytosis, the subsequent defects in FM1-43 destaining are likely to reflect problems in the recycling of vesicles, either within the RRP or between the reserve pool and the RRP. Whether this represents an ongoing role of type III Nrg1 back-signaling during transmitter release per se or is secondary to other deficits in the formation of active release sites cannot be distinguished at this time.

Quantification of the number of SV clusters, either by FM1-43 loading or at the ultrastructural level revealed significant decreases in type III Nrg1 heterozygous axons compared with wild type. Similar decreases were not seen when we quantified vesicle clusters by vGluT1 antibody staining. It is possible that antibody staining is identifying secretory vesicles that are trafficking along axons that have not yet contributed to formation of mature, functional SVs (Ahmari et al., 2000).

It has been shown before that presynaptic type III Nrg1 also is required for targeting of nAChRs to presynaptic sites at vHipp-nAcc, and at cortical-BLA synapses where they are important modulators of glutamate release (Zhong et al., 2008; Jiang et al., 2013). Here, we demonstrate an additional level at which Nrg1/ErbB4 signaling modulates neurotransmitter release; by regulating the vesicle recycling process.

## Conclusion

We conclude that at vHipp-nAccs synapses, presynaptic type III Nrg1 bidirectional signaling controls the establishment of vHipp-nAcc glutamatergic synapses. Type III Nrg1 back-signaling contributes to functional SV clustering and neurotransmitter vesicle release. Presynaptic type III Nrg1, either acting through postsynaptic ErbB4 receptors, or secondary to presynaptic glutamate release (or both) controls the extent of postsynaptic glutamatergic synapse formation and the synaptic recruitment of GluA2 AMPA receptors. Genetic perturbation of Nrg/ErbB signaling contributes to dysfunction of glutamatergic synaptic transmission through both presynaptic and postsynaptic mechanisms.

## Synthesis

Reviewing Editor: Zoltan Nusser, Institute of Experimental Medicine Decisions are customarily a result of the Reviewing Editor and the peer reviewers coming together and discussing their recommendations until a consensus is reached. When revisions are invited, a fact-based synthesis statement explaining their decision and outlining what is needed to prepare a revision will be listed below. The following reviewer(s) agreed to reveal their identity: Arlene Hirano, Ruud Toonen

The authors demonstrate that altered bidirectional type III neuregulin 1 (Nrg1)/ErbB signaling at ventral hippocampal-accumbens synapses results in changes in pre- and postsynaptic organization. A number of complementary techniques, including electrophysiological recordings, immunocytochemistry, FM 1-43 uptake and de-staining, and electron microscopy, have been used to assess synaptic changes as a consequence of reduced presynaptic Nrg1. One of the main conclusions is that synaptic vesicle clustering and FM1-43 de-staining was decreased in Nrg1 heterozygote neurons. The ability to reverse the reduction in functional synaptic vesicles in the Nrg1 het vHipp axons by exogenous application of ErbB4 is powerful. There are a number of issues the authors should address prior to publication.

Comment 1 concerning over interpretation of the data:

1a. In my opinion the title of the manuscript is not supported by the data: the authors did not assess vesicle clustering and trafficking at hippocampal-accumbens synapses but in axons from hippocampal neurons not connecting accumbens cells. This should be changed. (why not stick to the data: "Axonal type III Nrg1 controls glutamate synapse formation and GluA2 trafficking in hippocampal-accumbens connections"). Alternatively, the authors could perform the experiments in the hippocampal-accumbens synapses.

1b. In the abstract the sentence: "Reduced expression of axonal type III Nrg1 along vHipp projections also decreases functional synaptic vesicle clustering and vesicular trafficking to presynaptic vHipp axonal terminals, thereby decreasing the frequency of glutamatergic mEPSCs". This is an assumption; the authors did not measure mEPSCs in these connections. Please change.

Comment 2 concerning FM1-43 assays:

The data plotted in figure 2 B-D are normalized to initial fluorescence of the FM1-43 signal. Please include bars showing the initial fluorescence after K+-dependent loading in WT and Nrg1 +/- synapses. The typical example in figure 2A shows equal intensities in both types of synapses, which is unlikely given the fact that the decreased vesicle cycling will also affect the K+-dependent labeling of the terminals. Please comment on this in the manuscript. In addition please specify the location of the axons: are these FM1-43 positive terminals within the hippocampal slice (and therefore likely synapses) or FM1-43 puncta on isolated axons (and therefore not synaptic connections).

Comment 3 concerning vesicle clusters in figure 5:

Please comment on the nature of the vesicle clusters. The clusters do not look like synapses (no active zone, no postsynaptic counterpart). Could these be varicosities? Are these clusters the same clusters analyzed using FM1-43? Please add explanation of this in the manuscript.

Figures 2 and 3 are less convincing. The authors use FM1-43 to assess vesicle cycling in axons from hippocampal slices of Nrg1 +/- and WT controls. Why did the authors use a different synaptic connection from the hippocampal-nAcc connections measured in figure 1?

In Figure 1E, as each of the arrows appears to point out differently shaped structures, at this level of resolution, it is not clear what was counted as a synaptic contact. What was the criteria for counting a "synaptic contact": was the yellow co-localization taken to be (e.g. the leftmost arrow in both panels) and counted as a synaptic contact, or large clusters of vGluT1, similar to the FM 1-43 clusters in later figures, with PSD95 contacts? If the yellow co-localization determined a synaptic contact, was the fluorescence intensity levels of the individual vGluT1 and PSD95 immunolabeling similar?

From data in Figure 1F, the authors state that the level of GluA2 was decreased at the synapses formed. Were the ratios determined for individual synaptic contacts? Does the ratio of the surface to total GluA2 at individual synaptic contacts correspond to the overall ratio of surface to total GluA2 measured? For the neurite measurements, were the ratios determined for a standard length of neurite, or all neurites? Was the synaptic density even along the whole length of the neurites for both WT and het Nrg1 vHipp-accumbens preps? Fewer synaptic contacts, as in Fig.1E, would result in fewer clusters of GluA2 and a lesser overall ratio of surface GluA2 to total.

In the FM 1-43 experiments, was there a significant amount of photobleaching during the recordings?

In the EM experiments, was there difficulty in identifying the glutamatergic neurites? Are all of the neurites from the explant from glutamatergic cells?

The lack of n. accumbens target neurons in the FM 1-43 and EM experiments suggests a lack of ErbB4 back-signaling to both WT and het Nrg1 vHipp neurites from the explants. For instance, would exogenous ErbB4 also be expected to affect the WT neurites?

Authors might include a discussion of other presynaptic mechanisms changed in altered NRG1/ErbB4 signaling, such as at GABAergic synapses (Woo et al., 2007).

Minor points:

Figure 1C,D: please add number of neurons recorded

Figure 1E: typo 100 mm should be 100 μm

Figure 1F: typo in blue DAPI label.

P13 line 8 from bottom: vesicle should be vesicles

P14: microclises should be microslices

P14 last paragraph: that should be the

P15 one but last sentence: add 'of' in between organization and synaptic

More details, such as catalog numbers and RRIDs, about the genetically modified mice, antibodies, and software tools used are needed in the materials and methods.

For the statistical analyses, a statistical table and the actual P values should be included.
